# Transcranial Magnetic Stimulation in Parkinson’s Disease and Parkinsonian Syndromes: A Narrative Expert Review

**DOI:** 10.3390/life16020233

**Published:** 2026-02-01

**Authors:** Mariagiovanna Cantone, Manuela Pennisi, Rita Bella, Raffaele Ferri, Francesco Fisicaro, Giuseppe Lanza, Maria P. Mogavero, Aurora Palmigiano, Angelica Quercia, Mario Zappia

**Affiliations:** 1 Unit of Neurology, Policlinico University Hospital “G. Rodolico-San Marco”, 95123 Catania, Italy; 2 Department of Biomedical and Biotechnological Sciences, University of Catania, 95123 Catania, Italy; manuela.pennisi@unict.it; 3 Department of Medical and Surgical Sciences and Advanced Technologies “G. F. Ingrassia”, University of Catania, 95123 Catania, Italy; rbella@unict.it (R.B.); m.zappia@unict.it (M.Z.); 4 Oasi Research Institute-IRCCS, 94018 Troina, Italy; rferri@oasi.en.it (R.F.); glanza@oasi.en.it (G.L.); paola_mogavero@libero.it (M.P.M.); aquercia@oasi.en.it (A.Q.); 5 Primary Health Care Unit, Provincial Health Authority of Siracusa, 96100 Siracusa, Italy; drfrancescofisicaro@gmail.com; 6 Department of Surgery and Medical-Surgical Specialties, University of Catania, 95123 Catania, Italy; aura.palmigiano@gmail.com

**Keywords:** cortical excitability, neural plasticity, neuromodulation, movement disorders, Parkinson’s disease, parkinsonism, neurorehabilitation

## Abstract

Transcranial magnetic stimulation (TMS) is a non-invasive brain stimulation tool for investigating the neurophysiology of different neurological and neuropsychiatric disorders, including Parkinson’s disease (PD) and other parkinsonian syndromes and movement disorders. Briefly, TMS enables targeted stimulation of specific cortical regions through externally applied magnetic pulses, avoiding surgical intervention (as it occurs in deep brain stimulation) and making it a safe, repeatable, and well-tolerated approach. Over the past two decades, extensive research has explored the clinical utility of TMS in PD, with particular emphasis on motor cortex excitability, synaptic plasticity, and functional connectivity, which are central contributors to both motor and non-motor symptoms in PD patients. In addition, repetitive TMS and related stimulation paradigms have been shown to positively modulate cortical plasticity, i.e., the brain’s capacity to reorganize neural circuits, suggesting potential benefits for longer-term non-pharmacological management and rehabilitation protocols. More recently, studies have also investigated the role of TMS in atypical and secondary parkinsonisms, indicating that it may help characterize distinct neurophysiological abnormalities and provide symptomatic improvement in selected patients. This narrative expert review provides a comprehensive summary of TMS applications across the wide spectrum of parkinsonian syndromes, highlighting not only clinical potential, but also methodological limitations and future research directions.

## 1. Introduction and Basic Principles

Over the past years, there has been an increased interest in exploring the pathophysiology of movement disorders with neurophysiology studies, using a variety of techniques such as electroencephalography, electromyography, and non-invasive brain stimulation (NIBS). Among the NIBS techniques, transcranial magnetic stimulation (TMS) revealed its utility as a safe and painless method able to explore and monitor the brain excitability and functioning.

TMS was developed in 1985 by Barker and colleagues [1] and through the evaluation of the primary motor cortex (M1), it has given important pathophysiological and clinical insights into the integrity and function of the cortico-spinal tract in healthy subjects and in patients with neurological disorders or systemic diseases also affecting the nervous system [2,3,4]. A comprehensive review on the diagnostic utility of TMS has been recently provided [5]. TMS operates on the principles of Faraday’s law of electromagnetic induction. A transducing coil, connected to a high-voltage, high-current discharge system, generates a strong, rapidly changing magnetic field. When this coil is positioned tangentially to the scalp, the magnetic field penetrates the skull with minimal attenuation and induces a secondary electrical current in the conductive intracranial tissues. This induced electric field is oriented perpendicular to the magnetic field and flows in the opposite direction to the current in the stimulation coil [6].

### 1.1. Single Pulse Measures

A single TMS pulse delivered over the primary motor cortex (M1) at the optimal scalp location evokes motor-evoked potential (MEP) in muscles on the opposite side of the body. The operator can adjust the intensity of the current passing through the coil [6], as well as modify the frequency and interstimulus interval (ISI) of the pulses-parameters that play a critical role in determining the physiological effects of TMS [7].

The resting motor threshold (rMT), i.e., the minimal stimulation intensity required to elicit MEPs at rest, is considered as a global measure of cortical excitability, since it is an aggregate index of the excitability of the motoneurons membranes, neuronal inputs in cortical pyramidal neurons, spinal motoneurons, neuromuscular junctions, and muscle cells [8]. While the rMT is most often defined as the minimum intensity to elicit a MEP of ≥50 μV in at least half of consecutive trials in the relaxed target muscle, the active MT (aMT) is defined as lowest intensity required to elicit an MEP amplitude ≥ 200 μV during slight isometric tonic muscle contraction.

The MEP amplitude, defined as the peak-to-peak magnitude of the motor response, primarily reflects the excitability of motor output neurons in the cortex, as well as the functional state of nerve roots and peripheral motor pathways, all the way to the target muscle [7]. The amplitude of MEP expresses a compound index of the output cells excitability within the M1, motor axons, nerve roots, peripheral motor nerves, and muscles [7]. The magnetic input–output (IO) curve and MEP amplitude assess neurons that are less excitable or spatially distant from the center of target muscle representation in the M1 and assess the strength of corticospinal projections [9]. Conversely, MEP latency represents the time between the TMS pulse over M1 and the onset of the muscle response, encompassing conduction through the central and peripheral nervous systems, the neuromuscular junction, and the muscle itself.

CMCT, on the other hand, is calculated by subtracting the latency of MEPs elicited via spinal (motor root) stimulation from those evoked by cortical stimulation. This measure isolates the conduction time along the corticospinal tract, specifically from upper to lower motor neurons [10]. The peripheral motor conduction time can be estimated using also the F-wave method, as reflected by the following formula (F + M − 1)/2, where F represents the shortest F-wave latency, M is the distal motor latency and 1 ms represents the turnaround time for spinal motor neurons activated antidromically [11]. The latency of the MEP and the central motor conduction time (CMCT) are both indicators of the integrity of the corticospinal tract.

The cortical silent period (cSP), which refers to the electromyographic inhibition of the motor responses occurring after a suprathreshold stimulation of the M1 during a tonic voluntary contraction of contralateral muscles, is an index of the inhibitory intracortical circuit [12]. This parameter, which normally lasts a few hundred msec, is considered as a dynamic metric of a specific inhibitory intracortical circuitry [13], largely mediated by the gamma-aminobutyric acid (GABA)-B activity [14]. Muscle activation and hemisphere stimulation of the same side evokes the ipsilateral SP (iSP), which it is thought to be generated by transcallosal fibers projecting to contralateral GABAergic interneurons [15].

### 1.2. Paired Pulse Measures

With the advance of novel paradigms such as paired-pulse, TMS becomes an intriguing research tool to evaluate different aspects of cortex functionality as intracortical circuits, cortical connectivity, and neural plasticity [16]. Paired-pulse TMS paradigms allow the assessment of the short-interval intracortical inhibition (SICI) and the intracortical facilitation (ICF) phenomena [17]. This standard protocol involves delivering a conditioning stimulus (CS) at a subthreshold intensity, followed by a test stimulus (TS) at a suprathreshold level. By adjusting the intensity of the CS and the interstimulus interval (ISI) between the two pulses, researchers can assess various aspects of intracortical interneuronal function and interaction. When the ISI is between 1 and 5 milliseconds, the CS reduces the amplitude of the MEP, a phenomenon known as short-latency intracortical inhibition (SICI) [17]. At longer ISIs, from 7 to 20 milliseconds, the CS enhances the MEP response, referred to as intracortical facilitation (ICF) [18].

The activity of GABA-A interneurons is the most likely mediator of SICI [19], whereas the neurophysiology of ICF is more complex. It probably relates to the activation of a cortical circuit projecting upon cortico-spinal cells different from those preferentially activated by single-pulse TMS. ICF seems to be greatly mediated by excitatory glutamatergic interneurons [20]. Long-interval intracortical inhibition (LICI) is typically elicited by a suprathreshold CS followed by a suprathreshold TS at 50–200 ms ISI [21,22]. LICI appears to be mediated by GABA-B post-synaptic receptors [23].

Short-latency afferent inhibition (SAI) is a TMS protocol used to assess sensorimotor integration by measuring the inhibitory effects of peripheral afferent input on motor cortex excitability. First described by Tokimura and colleagues [24], the technique involves delivering an electrical stimulus to a peripheral nerve (typically the median nerve at the wrist), followed approximately 20 milliseconds later by a TMS pulse over the contralateral primary motor cortex. Under normal conditions, this results in a significant suppression of the MEP amplitude, reflecting an inhibitory process believed to be mediated by central cholinergic circuits. Accordingly, in normal conditions, scopolamine (a muscarinic antagonistic) abolishes or reduces SAI [25], whereas acetylcholine positively stimulates it [26]. Previous evidence suggests that SAI might reflect the integrity of intracortical circuitries underlying sensory-motor integration [27,28,29,30]. As such, SAI provides insights in disorders affecting cognition and movement [28]

### 1.3. TMS Neuroplasticity-Related Measures

Distinctive rTMS protocols, such as the paired associative stimulation (PAS) [31], the theta burst stimulation (TBS) [32], and quadripulse stimulation (QBS) paradigms [33], can aid toward a further understanding of the complex phenomena of neural plasticity including the so-called “metaplasticity” [34]. These methods reproduce the findings of in vitro and in vivo long-term potentiation (LTP) and long-term depression (LTD) protocols used in animal models and are commonly used to assess or induce LTP-/LTD-like plasticity in the healthy human M1 and in patients with movement disorders.

The PAS protocol implies an application of magnetic stimuli after a short time interval of exercise or by means of LF-repetitive electric stimulation of median nerve coupled with TMS of the contralateral M1. TBS is a type of patterned rTMS with several advantages, such as short time of single treatment and low stimulation intensity compared with traditional rTMS. However, TBS applied to the prefrontal cortex produced more variable responses, with no consistent patterns, maybe due to the individual participant and methodological factors [35].

### 1.4. Repetitive TMS Paradigms

The rTMS, in which trains of magnetic impulses are delivered in rapid succession on the same cortical target, allows to transiently modulate the functioning of the stimulated cortical area, also through the induction/release of neurotrophins and angiogenic factors, the change in electrocortical excitability, and the variation in cerebral blood flow (CBF), thus inducing short- and long-term neuroplastic phenomena. This mainly depends on the stimulation frequency, with high-frequency (HF) stimulations (>1 Hz) usually excitatory towards the stimulated cortical target, while low-frequency (LF) stimulations (≤1 Hz) are often inhibitory.

Although the mechanisms behind these neurobiological effects are not yet fully understood, they are thought to be related to processes such as LTD and LTP in the brain. rTMS has a broad spectrum of therapeutic and rehabilitative applications, and in 2008, it was approved by the Food and Drug Administration as an adjunct treatment for adults with drug-resistant major depression (MD) [36]. Subsequently, rTMS was applied to in other psychiatric and neurological conditions, including movement and motor disorders, as a possible therapy [37,38,39]. In this context, rTMS guidelines and their update were published [40,41].

### 1.5. TBS Paradigms

Theta-burst stimulation (TBS) is a form of rTMS that delivers patterned bursts of stimulation at a frequency mimicking the natural theta rhythm of the brain (5 Hz). TBS typically involves three pulses delivered at 50 Hz, repeated at intervals of 200 msec. It has been shown to induce LTP-like or LTD-like effects on cortical excitability depending on the frequency and the parameters used.

There are two commonly used patterns of TBS, continuous (cTBS) and intermittent (iTBS). In iTBS, twenty 2 s periods (ten bursts) of TBS are applied at a rate of 0.1 Hz. The iTBS, which enhances cortical excitability, has been shown to be beneficial in several animal models of neurodegeneration, including PD [42,43]. In cTBS, bursts of three pulses at 50 Hz are applied at a frequency of 5 Hz for either 20 s (100 bursts) or 40 s (200 bursts). cTBS reduces cortical excitability and it has been used to modulate overactive brain areas. TBS offers the advantage of a shorter stimulation duration compared to conventional rTMS protocols, making it more practical for clinical applications. Moreover, TBS has been shown to influence network connectivity and functional reorganization in the brain, further enhancing its potential as a therapeutic tool. Given its promising results, TBS is increasingly being explored as an effective treatment modality for various neurological disorders.

### 1.6. QPS Paradigm

Quadripulse stimulation (QPS) is a patterned form of repetitive transcranial magnetic stimulation (rTMS) designed to induce long-lasting changes in cortical excitability. Introduced by Hamada et al. [44], QPS delivers bursts of four monophasic magnetic pulses at fixed interpulse intervals, typically repeated every 5 s for a total duration of 30 min. The direction and magnitude of the induced plasticity, i.e., LTP-like or LTD-like depend on the specific interpulse interval used: shorter intervals (e.g., 5 msec, QPS5) tend to facilitate cortical excitability, while longer intervals (e.g., 50 msec, QPS50) are associated with inhibitory effects. QPS has demonstrated superior reliability and interindividual consistency in modulating motor cortex excitability compared to conventional rTMS protocols, making it a valuable tool for probing synaptic plasticity in both healthy individuals and clinical populations. Moreover, due to its precise temporal structure and robust aftereffects, QPS has been increasingly employed in the study of neuroplastic alterations in movement disorders, including PD and atypical parkinsonian syndromes.

## 2. Methods

This article was conceived as a narrative expert review rather than a PRISMA-compliant systematic review. Although a systematic approach would have provided a more in-depth analysis of the available evidence, a narrative review has been considered the most appropriate framework at this stage to integrate neurophysiological, clinical, and translational perspectives. Because of this, the marked heterogeneity of TMS paradigms, stimulation targets, outcome measures, and patient phenotypes across the wide spectrum of parkinsonian syndrome are considered here.

The literature search was conducted using PubMed, focusing on peer-reviewed studies investigating TMS in PD and other parkinsonian syndromes. Keyword combinations included “transcranial magnetic stimulation”, “motor cortex excitability”, “Parkinson’s disease”, and “parkinsonism”. Reference lists of relevant articles were also screened. Animal studies, as well as non-English written papers, were excluded. Unlike PD, sections on atypical and secondary parkinsonsims are intended as exploratory and hypothesis-generating, rather than as a comprehensive or definitive review.

## 3. TMS Insights into Parkinson’s Disease Pathophysiology

PD is a progressive neurodegenerative movement disorder affecting approximately ~1% of the population over 65 years [45]. Although the hallmark histological lesion is the degeneration of the substantia nigra pars compacta (SNc), the neuropathology extends across several regions. Thinning of the medial frontal (premotor and supplementary) motor cortex, posterior cingulate cortex, precuneus, lateral occipital and temporal cortex, as well as the dorsolateral prefrontal cortex (DLPFC), has been documented [46]. Motor features such as tremor, bradykinesia, rigidity, and postural instability are the hallmarks for its clinical assessment and diagnosis [47].

Traditionally, motor symptoms are used for PD subtyping, with tremor-dominant and postural instability and gait difficulty as the most consistently identified motor subtypes [48]. However, PD is highly heterogeneous, encompassing complex motor and non-motor clusters [49]. More recently, a “mild motor-predominant” subtype (mild motor and non-motor symptoms) or cluster I and a “diffuse malignant” subtype (combination of severe motor and non-motor manifestations) or cluster II were identified [50]. The classical model of PD pathophysiology, centered on dopaminergic depletion in the SNc, leads to functional alterations within the basal ganglia–thalamo–cortical (BGTC) circuits. TMS provides a non-invasive “looking glass” into these systemic changes.

### 3.1. Basic Cortical Excitability Measures: Motor Thresholds and Recruitment Curves

The assessment of rMT and aMT in PD reveals heterogeneous results in cortical excitability patterns, reflecting the complex interplay between nigrostriatal denervation and compensatory cortical reorganization. Although most early studies often showed normal rMT [51,52,53,54,55] and aMT [56], other studies points toward a state of cortical hyperexcitability [57,58,59], particularly on the side exhibiting greater rigidity [60,61,62]. Overall, PD patients with tremor-dominant subtype exhibit reduced rMT and aMT compared to akinetic-rigid patients [57]. Another recent pilot study reported a reduction in M1 excitability, reflected by an increased resting motor threshold (rMT), both during freezing episodes and in the transitional period preceding them, suggesting that altered M1 excitability may play a critical role in the pathophysiology of freezing in PD [63].

From a mechanistic standpoint, this hyperexcitability may represent a compensatory response: the motor cortex increases its baseline excitability to counteract the pathological “braking” effect exerted by the overactive internal globus pallidus (GPi) on the thalamus. This is supported by the increased MEP amplitude and steepness of the IO curve [59], which correlates with bradykinesia. As the disease progresses, the motor system loses its “dynamic range”, operating at a higher but less flexible level of excitability.

Conversely, CMCT remains largely preserved in the early stages of idiopathic PD, suggesting that the corticospinal tract is structurally intact [51,52,64,65]. However, significant shortening of CMCT in late-stage disease [66,67] may reflect a loss of inhibitory control over fast-conducting pyramidal neurons, with CMCT that might be prolonged in levodopa-PD patients [65,66,67]. However, prolonged CMCT was consistently found in early onset PD patients with *Parkin* mutation (PARK2) indicating that specific genetic backgrounds may involve primary pyramidal dysfunction [64,68,69].

### 3.2. Contralateral and Ipsilateral Silent Period

The cSP is consistently reduced in PD [70], thus providing neurophysiological evidence of impaired cortical inhibitory tone. Dopaminergic therapy [71] and deep brain stimulation (DBS) of the internal globus pallidus (GPi) [72] tend to normalize cSP duration. Of further relevance, a significant reduction in cSP duration has been reported in PD patients that were in the “off” compared to “on” state, although in both states the cSP duration was not significantly different when compared to healthy controls [55].

Consequently, monitoring cSP duration could serve as a therapeutic biomarker in PD, although its high inter-individual variability remains hard to introduce for routine clinical stratification. Moreover, PD patients with tremor-dominant subtype have shorter iSP duration, a marker of the function of the corpus callosum, compared to akinetic-rigid patients, while iSP latency tended to be longer in akinetic-rigid patients compared to healthy controls [57]. The iSP was shorter and smaller with stimulation of the more affected hemisphere but could be restored by levodopa [62].

### 3.3. Intracortical Inhibition and Facilitation

One of the most robust TMS findings in PD is the impairment of intracortical inhibitory circuits. SICI is constantly reduced [53,54,55,56,73,74] even in early, drug-naïve stages [73]. With disease progression, there is a further reduction in SICI [75]. The fact that dopaminergic medication [54,55] and DBS of the subthalamic nucleus (STN) [76] can restore SICI suggests that this GABAergic deficit is clearly dopamine-dependent and functionally linked to the BGTC loop’s imbalance.

While SICI is a robust marker, LICI has yielded highly inconsistent results in PD: contradictory reports of reduced [77,78], normal, or even increased LICI have been reported [30,79,80], highlighting a significant limitation in the current literature caused by the high sensitivity of LICI to experimental parameters (such as conditioning stimulus intensity and ISIs). This lack of methodological standardization precludes the use of LICI as a reliable diagnostic biomarker at this stage. SICI and LICI are both measures of cortical inhibition in the human brain; however, the reduction in SICI may be caused by a dysfunction in fast-acting GABA-A receptors or the low-threshold interneurons that release GABA-A [81]. While LICI depends on high-threshold interneurons and slower GABA-B receptors, these circuits often remain functional even when the SICI circuit is impaired [23,82].

Similarly, the findings on ICF in PD produce varibale results, with some studies showing reduced ICF [83,84] and others finding normal ICF [55,80]. The increase in SICF was observed in de novo PD patients [85] and is further enhanced in PD patients with dyskinesia [86]. In another study, the combined effect of SICI and SICF (ISI 1.5 msec) was comparable between drug naïve PD patients and healthy controls [85]. Interestingly, patients with idiopathic REM-sleep behavior disorder (RBD) and those with both PD with RBD shared a reduced ICF, suggesting an early glutamatergic dysregulation before the onset of conventional parkinsonian motor symptoms [52].

Mechanistically, this suggests a failure of the basal ganglia’s “filter” function, which leads to abnormal tonic excitatory drive to the motor cortex and eventually manifesting as a loss of the cortical inhibitory tone necessary for fine motor control.

### 3.4. Short-Latency Afferent Inhibition

Reduced SAI, especially on the more affected side [30], is a stable finding in PD with cognitive deficits [28,87,88,89,90], confirming the involvement of cholinergic dysfunction in the development of cognitive impairment in PD. Reduction in SAI in PD patients seems associated with a higher risk of developing cognitive decline and other associated symptoms, including visual hallucinations [91], dysphagia [92], olfactory dysfunction [93,94], and RBD [88]. These findings suggest that SAI could potentially serve as a biomarker for PD cognitive decline. Interestingly, these results strongly implicate SAI abnormalities and visual hallucinations as two epiphenomena of the cholinergic system dysfunction in PD patients who will probably develop dementia.

Neurophysiological studies revealed stronger that SAI is associated with higher gait speed and longer step length in patients receiving dopaminergic medications [89], while reduction in SAI was also evident in PD patients prone to falling, even after adjusting for cognitive function [95], suggesting a role for SAI as a predictive biomarker for gait, posture, and balance impairment. Neuroimaging studies has documented structural and functional abnormalities in a number of cortical and subcortical brain regions including cholinergic deficits in PD patients with freezing of gait (FoG) [96]. Despite the presence of cognitive deficits (poorer executive and visuospatial performances), a study failed to detect any significant decrease in cholinergic activity evaluated by SAI in FoG+ compared to FoG- [97]. However, in a more recent study, reduced SAI was associated with severe FoG manifestations, impaired gait characteristics and variability in PD patients with ON-OFF-FoG, suggesting impaired thalamocortical cholinergic-GABAergic SAI pathways underlying ON-OFF-FoG [98].

### 3.5. TMS-Derived Neural Plasticity in PD

The ability of the brain to modify synaptic strength (LTP-/LTD-like plasticity) is severely hampered in PD, as clearly demonstrated by abnormal responses to PAS [99,100,101] and TBS protocols [102,103,104]. Interestingly, this deficit is asymmetric as observed in the more affected side, whereas the less affected side was characterized by exaggerated responses interpreted as a compensatory mechanism [105] and such asymmetry progressively decreased over time [75]. A study has also suggested a correlation between MEP changes elicited by PAS and the likelihood of developing early motor complications [106]. Interestingly, the achievement of a sustained long-duration response (LDR) to levodopa may act synergistically with motor learning to induce adaptive changes in neuroplasticity in basal ganglia and cortical networks measured by MEPs [107].

However, a study revealed that iTBS produced similar effects on cortical excitability in PD patients and in controls [108]. In the study of Belvisi and colleagues [109] comparing the “mild motor-predominant” subtype to the “diffuse malignant” subtype, reduced responses to iTBS were observed in the “diffuse malignant” subtype. These data may indicate that the subtyping of PD patients is not a mere clinical classification but reflects different pathophysiological mechanisms and could represent promising biomarkers to evaluate PD subtypes.

A schematic representation of cortical excitability and plasticity measures, highlighting their role in diagnosis, monitoring, and treatment of PD is shown in Table 1.

## 4. Experimental Role of rTMS, TBS, and QPS Protocols in PD

### 4.1. Repetitive TMS (rTMS)

Recently, TMS has received attention as a possible non-invasive neuromodulatory treatment for PD, with an increasing number of studies exploring the therapeutic effect of protocols such as rTMS and TBS on both motor and non-motor symptoms. Although the effects of TMS are restricted to the cortex, stimulating appropriate cortical regions within the basal ganglia circuitry can modulate activity within these loops, potentially offering clinical benefits [110]. Following the pioneering work of Strafella and colleagues [111] and Kim and colleagues [112], numerous studies have supported the idea that modulating cortical activity directly through rTMS can produce secondary effects on interconnected structures within the basal ganglia-cortex loops. However, these studies vary in terms of stimulation protocols and cortical targets, such as the M1 and the prefrontal cortex.

While one study found no significant difference between “sham” (ficitious) and “active” (real) rTMS [113], several studies suggest a consensus regarding the efficacy of HF-rTMS on M1 for motor symptoms and FoG [40,112,114,115,116,117,118,119]. This efficacy increases with bilateral stimulation or potentiation via electrical stimulation of the leg cortical representation or hand representation [120,121]. These studies reported improvement in the UPDRS-III scores ranging from 15% to 26%, with effects persisting for several weeks up to one month [112,119,120,121], as confirmed by various meta-analysis [122,123,124]. A systematic review highlighted the beneficial effects of rTMS on FoG and cognitive dysfunction in PD [125]. Evidence indicates that both 1 Hz and 25 Hz rTMS outperformed sham stimulation in reinforcing activity-dependent plasticity, leading to sustained improvements in motor symptoms and dual-task walking [126]. Additionally, the positive impacts on depressive symptoms and health-related quality of life were observed following HF bilateral M1 rTMS [121].

The supplementary motor area (SMA) remains a critical target due to the fact that its dysfunction contributes to motor symptoms such as bradykinesia, freezing of gait, and difficulty in sequential movement. However, studies have yielded mixed results regarding the effects of SMA stimulation. While some research reported positive outcomes [127,128], others observed less favorable results [129]. Improvements in the UPDRS-III score were modest, ranging from a 4.5% to 6.8% reduction [127,128], although SMA has been identified as a highly suitable target for PD patients with FoG [130].

Recently, 5 Hz TMS targeting the left SMA once per week for 8 weeks demonstrated significant motor improvements, as evidenced by the enhancement in clinical scales score, dexterity performance, and alterations in connectivity with remote brain regions, such as the right precentral area, superior frontal gyrus, middle frontal gyrus, thalamus, and cerebellum [131]. Chi et al. [132] corroborated these findings, showing that patients with lower sensorimotor connectivity experienced greater improvement.

Other targets include the cerebellum; LF (1 Hz)-rTMS targeting the medial or lateral cerebellum has shown promise for tremor, that is often resistant to dopaminergic therapy [133]. Minks et al. [134] demonstrated that 1 Hz cerebellar rTMS applied to the right hemisphere influenced upper limb motor performance in early-stage PD patients in a differential manner, improving gross motor skills while impairing fine motor skills.

Regarding depression, 15 Hz-rTMS showed non-inferiority compared to fluoxetine [135] and stimulation of the left DLPFC produced beneficial effects lasting at least 30 days [136]. Meta-analyses confirm that DLPFC-rTMS improves depression similarly to treatment with selective serotonine reputake inhibitors [123,137,138]. These findings are supported by a recent systematic review, which reported that NIBS, particularly rTMS, effectively alleviated depression and depressive symptoms in PD patients when compared to sham stimulation or placebo. However, no improvement was observed in anxiety or apathy; also, no significant difference was found between NIBS and antidepressant therapy alone [139]. Finally, Yokoe et al. [140], in the attempt to determine whether which of the three consecutive days of HF-rTMS over M1, SMA, and DLPFC were the best treatment targets, showed that while HF-rTMS over M1 and SMA improved motor symptoms, it did not alter mood disturbances.

### 4.2. Theta Burst Stimulation (TBS)

Notably, iTBS has been shown to increase striatal dopamine levels in hemiparkinsonian rats and reduce astrocytic and microglial activation [141]. In animal models of PD, iTBS has demonstrated improvements in both motor and behavioral functions by modulating the expression of NMDAR subunits [43], reducing oxidative stress markers, and increasing antioxidant levels [142]. Additionally, iTBS has been found to restore the balance between dopamine and adenosine signaling [143]. These findings led to double-blind randomized clinical trials aimed at investigating the plasticity induced by iTBS in PD patients and its potential rehabilitative applications [32,144,145].

Promisingly, bilateral iTBS over M1 has been associated with significant clinical benefits in postural stability, gait disturbances, and limb bradykinesia, as well as enhancements in UPDRS part III scores, accompanied by biological changes such as reduced GFAP levels, increased BDNF expression, and enhanced functional connectivity in the putamen–parietal–cerebellar and SMA–prefrontal networks [146]. A meta-analysis reported that cTBS over the SMA improved UPDRS-III scores in the OFF state [147]. Additionally, combining iTBS over the SMA with video game-based training improved manual dexterity [148]. Currently, the NET-PD study [149] is investigating the long-term neuroprotective potential of iTBS [150]. For dyskinesia, cerebellar cTBS has shown anti-dyskinetic effects [151], confirmed by a reduction in serum BDNF [152]. Conversely, TBS over the dorsal pre-motor cortex did not significantly interfere with dexterity [153].

TBS evidence in PD is growing, but studies often fail to investigate its impact in relation to dopaminergic medication status [147]. Only a limited number of studies have assessed TBS in ON [134,153] or OFF state [102,154], while others evaluated both [155,156,157,158]. As such, findings remain inconclusive, potentially due to the dampening effect of dopaminergic drugs on plasticity and the insufficient number of stimulation sessions.

### 4.3. Quadripulse Stimulation (QPS)

Hamada et al. [44] demonstrated that QPS-induced plasticity was significantly attenuated in PD, particularly evident when using a facilitatory protocol. Subsequent works [159,160] showed that dopaminergic medication partially restored this plasticity, supporting the potential of QPS as a biomarker of cortical dysfunction, although further large-scale and longitudinal studies are warranted.

Despite the therapeutic promise of rTMS and TBS in PD, several methodological challenges limit clinical translation. First, there is significant protocol heterogeneity regarding stimulation frequency, total pulse count, and the number of sessions, which complicates the definition of standardized clinical guidelines. Second, the interaction with dopaminergic medication remains a confounding factor; the ON vs. OFF medication state significantly alters cortical plasticity, with some evidence suggesting that dopaminergic drugs may dampen the expected plastic response to TBS. Finally, the majority of studies are limited by small sample sizes, highlighting the urgent need for large-scale, multicenter longitudinal trials, such as the ongoing NET-PD study. Moreover, despite some promising outcomes, it is crucial to note that the efficacy of rTMS is highly dependent on the disease stage and medication state. Many studies reporting positive effects are limited by a small sample size and a lack of long-term follow-up.

Consequently, as a whole, while TMS shows potential as a symptomatic add-on therapy, it cannot yet be considered a first-line treatment in clinical guidelines.

## 5. TMS and Drugs in PD

Long-term therapy with levodopa and dopamine agonists in PD patients is complicated by the development of fluctuations in motor response, such as levodopa-induced dyskinesia (LID). One Hz rTMS over the SMA reduced drug-induced dyskinesia by continuous apomorphine infusion in a group of patients with advanced PD [161].

Chronic levodopa intake seems to improve the abnormal PAS-induced plasticity in PD [101,162] but not the iTBS-induced LTP plasticity [103,104] with similar no effect of acute L-DOPA administration in de novo PD patients [104]. Different plasticity responses to levodopa according to the patients’ clinical features (stable responders to levodopa, motor fluctuations without LID, and motor fluctuations with LID) have been demonstrated by several studies [104,163,164]. Of note, a previous work on healthy subject demonstrated that L-Dopa enhanced both LTP- and LTD-like plasticity as compared to placebo. In contrast, neither an LTP- nor LTD-like effect was modulated by pramipexole highlighted that both D1 and D2 coactivation is required for LTD [159]. These data should be taken into consideration when enrolling patients with complex therapies.

Most studies have demonstrated reduced LTP-/LTD-like plasticity in patients with PD, with possible beneficial effect of levodopa in modulating the response to TBS and PAS protocols [165]. Recently, a study described that QPS-induced LTP-like effect was reduced in PD and restored by L-DOPA. The degree of the LTP was negatively correlated with MDS-UPDRS I and III scores, but not with MMSE and MoCA-J. Interestingly, the upper limb bradykinesia and rigidity showed a negative correlation with the LTP-like effect whereas the tremor had no correlation [166]. In patients with advanced PD, switching from intermittent to continuous levodopa delivery increased corticospinal excitability and improved deficient intracortical inhibition and abnormal motor cortex plasticity, along with amelioration of motor fluctuations and dyskinesia [167].

So far, data on the utility and efficacy of TMS seem to provide encouraging perspectives on the diagnostic and therapeutic applications of NIBS techniques. Some TMS parameters in PD showed differences in laterality or are more affected in phenotypes with a more aggressive course. Thus, it would be useful and desirable to identify stable and reproducible profile that allow the neurophysiological characterization of clinical phenotypes. This, moreover, could allow expanding the therapeutic role of NIBS in PD and other movement disorders.

As rTMS technologies advance, navigated rTMS for NIBS of motor and non-motor areas emerged. Using MRI scans, this technology allows greater accuracy and precision in the stimulation site. Moreover, the same area can be stimulated in each consecutive session over the entire treatment course that reduces variability of responses. These features make the navigated systems much more reliable and user-friendly, compared with regular hand-held coils equipment. Despite the potential advantages of navigated rTMS, its efficacy has not been tested extensively yet.

## 6. TMS and Sleep Disorders in PD

Recently, some studies explored the use of TMS to address diagnostic and therapeutic issues related to sleep disorders in PD patients. This is of particular clinical and research interest, given the common prevalence (e.g., insomnia) and the strong predictive role (i.e., RBD) of some sleep disorders in PD patients, even before the onset of typical motor symptoms. Indeed, TMS alterations commonly found in these patients may contribute not only to motor but also to non-motor symptoms, including sleep disturbances. Interestingly, these might correlate with the severity of motor symptoms that can disrupt sleep, such as rigidity and bradykinesia, thus hypothesizing a complex bidirectional intersection between motor and sleep disorders in PD [168]. Although the direct application of TMS in PD-related sleep disorders is less common, it can help to understand the pathophysiology underlying both clinical manifestations and can be integrated with other techniques, such as advanced neuroimaging [169].

In this scenario, RBD, i.e., a loss of physiological inhibition of muscle tone during REM sleep characterized by dream-enacting behaviors, is widely recognized as a prodromal manifestation of alpha-synucleinopathies. Patients with isolated RBD (iRBD) have an extremely high estimated risk to develop a neurodegenerative disease, even after a long follow up [170]. Moreover, in comparison with PD patients without RBD, the occurrence of RBD seems to identify a unique, more malignant PD phenotype, characterized by more severe motor and non-motor symptoms and increased risk for cognitive decline [171,172]. While some medications (e.g., melatonin, clonazepam, etc.) may have some therapeutic benefits on RBD, there is no available treatment able to modify the disease course or, at least, slow down the neurodegenerative phenoconversion [173]. Nevertheless, the long prodromal phase may allow an early diagnostic and therapeutic window and, therefore, the identification of any TMS marker of disease onset and progression is crucial [149].

To date, however, only few studies have explored the TMS correlates of RBD and its progression. The first study in RBD found an impaired SAI, which supported the cholinergic dysfunction in those patients developing cognitive decline [174]. This was further supported in RBD patients in the context of PD [88]. As such, cholinergic dysfunction may substantially contribute to non-motor aspects of PD as well, raising also the hypothesis that RBD increased the risk of cognitive changes in PD. A more recent study found that iRBD patients exhibited an electrocortical profile similar to that observed in PD [152]. Moreover, intracortical disinhibition was related to muscular tone change, which supports the model of retrograde influence of the brainstem to the cortex [175]. Therefore, an altered control in RBD, which would arise from the brainstem and ascend to the cortex, may determine both a reduced atonia during REM sleep and an imbalanced cortical disinhibition and hypofacilitation, favoring the former. Recently, a direct comparison between iRBD and PD with RBD revealed that both patient groups had a significantly decreased intracortical facilitation compared to healthy controls, thus sharing the involvement of glutamatergic transmission [52]. Finally, another report has recently compared PD without and with RBD: an enhancement of GABA-mediated and a reduction in glutamine-mediated activity was found in PD+RBD only, thus suggesting a distinctive pathophysiological processes in these subjects [176].

Several studies have investigated the therapeutic potential of TMS for sleep disorders in PD. For instance, very recently, Khedr et al. found that rTMS applied over the parietal cortex improved sleep quality in PD patients, reducing sleep fragmentation and nocturnal awakenings [177]. Another recent study explored the effects of rTMS on excessive daytime sleepiness (EDS) in PD, demonstrating that LF-rTMS over the right dorsolateral prefrontal cortex (DLPFC) may alleviate EDS symptoms [178]. Furthermore, it has been found that HF-rTMS over the parietal cortex improved sleep quality; in particular, rTMS improved sleep fragmentation and sleep efficiency, and reduced nocturnal awakenings [179]. Lastly, to date, no study has applied rTMS in iRBD or in PD patients with RBD, as recently reviewed [180]. Overall, studies suggest that TMS can be a potential therapeutic intervention for sleep disorders in PD. Stimulation parameters and target areas need to be carefully used to address various sleep-related symptoms; similarly, adequate monitoring is needed for adequate follow-up.

## 7. TMS in PD-Associated Dementia

Although PD was historically regarded primarily as a motor disorder, it is now well recognized that behavioral, personality, and cognitive changes, culminating in dementia, are major non-motor features of the late stages of the disease [181]. Since most TMS studies in PD have excluded patients with overt dementia, available insights on PD-related dementia (PDD) are often inferred from subgroup analyses involving individuals with mild cognitive impairment (MCI) or early cognitive symptoms. Among the neurophysiological measures, SAI—which is thought to reflect central cholinergic function—has emerged as particularly relevant. SAI is significantly reduced on the more affected side in PD patients overall [30] and is markedly diminished in those with dementia [87,182] or MCI [88,90,91]. These findings suggest that abnormal SAI may represent an early biomarker of cholinergic dysfunction and may be associated with the development of visual hallucinations, both of which are considered clinical harbingers of cognitive decline and eventual dementia in PD, as previously introduced.

A recent pilot study explored the effects of HF-rTMS over the M1 in patients with PD and comorbid dementia. The authors reported improvements in motor performance and modest gains in cognitive scores (MMSE, MoCA), suggesting a potential role for M1-targeted stimulation even in cognitively impaired patients [183]. Another study evaluated the effects of intermittent theta burst stimulation (iTBS) over the left dorsolateral prefrontal cortex (DLPFC) in patients with PD-MCI. Significant improvements were observed in visuospatial and executive functions, with effects persisting up to one month post-treatment [184]. In contrast, another randomized controlled trial failed to find significant cognitive improvements following bilateral HF-rTMS over the DLPFC in a similar population, highlighting the variability in responsiveness and the potential dependence on baseline cognitive network integrity [185].

## 8. TMS in Atypical Parkinsonian Syndromes

While TMS has been extensively studied in idiopathic PD, its application in atypical parkinsonian syndromes, such as PSP, MSA, CBD, and dementia with Lewy bodies (DLB), remains relatively underexplored. These disorders, characterized by distinct pathophysiological mechanisms and clinical manifestations, often exhibit limited and transient responsiveness to conventional dopaminergic therapies, highlighting the need for alternative therapeutic approaches.

Preliminarly, unlike TMS in idiopathic PD, which is supported by a large and methodologically mature body of evidence, data in atypical and secondary parkinsonisms remain sparse, heterogeneous, and largely exploratory.

Table 2 provides a summary of TMS changes and mechanistic interpretations to facilitate the differential diagnosis between PD and atypical parkinsonian disorders.

### 8.1. Progressive Supranuclear Palsy

PSP is a neurodegenerative disorder clinically characterized by akinetic rigidity, early-onset gait instability, axial dystonia, and impaired voluntary vertical gaze. In rare cases, PSP may initially present with features resembling primary lateral sclerosis, without typical parkinsonian signs, gaze palsy, aphasia, or dementia. In such cases, post-mortem analysis has revealed classical PSP-related neuropathology involving motor regions. Specifically, tau-positive, argyrophilic tufted astrocytes, neurofibrillary tangles, coiled bodies, and thread-like processes have been observed in M1 and superior frontal gyrus, with lesser involvement of the basal ganglia and brainstem nuclei. Marked fibrillary gliosis has also been documented in the precentral gyrus and corticospinal tract [186]. Based on these considerations, a TMS-based exploration of PSP may disclose valuable diagnostic and prognostic hints, along with some experimental treatment approaches.

Patients with PSP included in TMS studies were diagnosed according to the NINDS criteria, although the samples varied in terms of disease stage and pharmacological treatment. A consistent finding across studies is impairment of interhemispheric callosal connectivity, assessed via the ipsilateral silent period (iSP), which may aid in the differential diagnosis of parkinsonian syndromes [187,188,189]. In addition to prolonged iSP, PSP patients have shown abnormally increased MEP amplitudes at rest [187] and a significant reduction in SICI, suggestive of reduced intracortical inhibition and M1 disinhibition [187,190,191].

Reduction in the CSP has been reported in a subset of eighteen patients [187,191], further supporting cortical disinhibition in PSP. Moreover, cTBS, which typically suppresses cortical excitability, paradoxically facilitated MEPs in PSP patients [191], again indicating altered plasticity or homeostatic regulation in M1. Lastly, cerebellar brain inhibition (CBI), a measure of functional cerebellar-cortical connectivity mediated by inhibitory projections, was found to be significantly reduced, reflecting impaired cerebellar modulation of cortical output in PSP [190,192], suggesting the involvement of Purkinje cells or the dentato–thalamo–cortical pathway. Interestingly, cerebellar iTBS improved dysarthria and enhanced cerebellar interhemispheric functional connectivity [190].

In PSP, preliminary studies have investigated the potential of cerebellar HF-rTMS to enhance CBI, aiming to improve postural stability and speech functions. A pilot study demonstrated that 10 Hz cerebellar rTMS increased CBI by 32–50% and yielded improvements in postural control and speech in two patients with PSP, suggesting a promising avenue for intervention [193]. Additionally, a double blind, placebo-controlled, crossover study involving twenty PSP patients applied HF-rTMS over the primary motor cortex and prefrontal areas during passive leg cycling. The study reported improvements in motor performance and apathy, indicating potential benefits of rTMS in PSP [194].

### 8.2. Multiple System Atrophy

Multiple system atrophy (MSA) is a progressive neurodegenerative disorder characterized by parkinsonism, autonomic dysfunction, cerebellar ataxia, and corticospinal tract involvement. Clinical subtypes of MSA are classified based on the predominance of symptoms as follows: MSA with cerebellar features (MSA-C), MSA with predominant parkinsonism (MSA-P), or MSA with prominent autonomic failure. A pathological hallmark of MSA is the presence of oligodendroglial cytoplasmic inclusions, which are frequently distributed across the primary and higher-order motor cortices, as well as the pyramidal, extrapyramidal, and cortico-cerebellar systems [195].

In patients with sporadic olivopontocerebellar atrophy, often associated with MSA, elevated MTs and reduced MEP amplitudes have been correlated with cerebral hemispheric atrophy [196]. CMCT to the lower limbs has been found to be prolonged, particularly in patients with longer disease duration [197,198]. However, the degree of corticospinal involvement remains somewhat controversial. In a study of forty-five patients with either MSA-C or MSA-P, MEPs were found to be within normal limits [199], suggesting preserved corticospinal conduction. In contrast, another study employing the triple stimulation technique, a more sensitive method for assessing corticospinal dysfunction, revealed corticospinal impairment in six MSA patients [200].

TMS investigations also point to a reduction in intracortical inhibition in MSA [187,201,202]. Specifically, SICI was significantly reduced at an ISI of 3 msec in 10 MSA-P patients, but not in 4 MSA-C patients, while ICF at ISI 12 msec was not significantly altered [201,202]. Interestingly, acute L-DOPA administration enhanced SICI in MSA-P patients without producing concurrent clinical improvement [201]. Additionally, SAI, a marker of cholinergic and sensorimotor integration, was abnormally reduced in 8 out of 10 MSA-P patients, indicating a disruption in sensory-motor cortical processing [203]. Cortical plasticity mechanisms also appear to be impaired in MSA-P: in contrast to the facilitatory effect observed in healthy individuals, 5 Hz repetitive TMS produced inhibitory effects on M1 excitability [204]. Furthermore, TBS, both intermittent and continuous, failed to modulate MEP amplitude in MSA-P and MSA-C, suggesting a strong deficit in M1 LTP/LTD-like plasticity [202].

Regarding MSA, the literature on rTMS application is scarce. Pan et al. [205] conducted a randomized, double-blind, sham-controlled trial to evaluate the effects of HF-rTMS on fatigue in patients with MSA. The study involved twenty-two patients who received either active rTMS (10 Hz, ten sessions) over the left DLPFC or sham stimulation. The primary outcome was fatigue severity, assessed using the Fatigue Severity Scale. Results showed a significant short-term improvement in fatigue levels in the active rTMS group compared to the sham group. Additionally, improvements in depression and sleep quality were observed. The authors concluded that HF-rTMS over the left DLPFC might offer a safe and effective short-term intervention for fatigue in MSA, potentially via modulation of non-motor circuits.

### 8.3. Corticobasal Degeneration

Corticobasal degeneration (CBD) is a rare neurodegenerative disorder that typically presents with asymmetric akinetic-rigid syndrome, which is poorly responsive to L-dopa, along with upper limb apraxia often associated with dystonia, commonly referred to as “alien limb” phenomenon. As the disease progresses, additional features such as progressive myoclonus, gait disturbances, ocular motility abnormalities, and a frontal-type dementia may emerge. Structural imaging studies have revealed atrophy and gray matter loss primarily affecting the premotor cortices and SMA [206,207].

Converging neurophysiological evidence suggests a functional impairment of motor areas in CBD. Resting MT is significantly elevated in CBD patients compared to healthy controls [187], indicating decreased cortical excitability. This hypoexcitability may be associated with the focal degeneration of cortico-cortical inhibitory circuits, potentially contributing to the characteristic motor impairments seen on the affected side. Some TMS evidences have reported shortening of cSP [208,209,210], along with disrupted interhemispheric inhibition (iSP), reflecting structural and/or functional callosal damage [189,211]. These alterations have also been correlated with cognitive deficits [212] and appear to worsen in parallel with the progression of motor symptoms [208].

Paired-pulse TMS studies targeting short ISIs between 1 and 5 msec have shown a reduction in SICI in the M1 contralateral to the clinically affected side, indicating further impairment of inhibitory interneuronal circuits [211,213,214]. Overall, CBD is characterized by increased MT, shortened cSP, reduced SICI, and impaired transcallosal inhibition. These findings point toward a widespread breakdown of inhibitory mechanisms within the motor cortex, likely attributable to progressive neuronal degeneration [215]. This global disinhibition may underlie the observed corticospinal and interhemispheric hypoexcitability, a hallmark of CBD [187].

Interestingly, motor mapping studies using TMS have demonstrated an expanded cortical representation of the hand area in CBD patients with alien-hand syndrome, which may represent a compensatory reorganization of motor networks in response to ongoing neuronal loss [216]. To date, no study has explored rTMS/TBS in patients with CBD.

### 8.4. Dementia with Lewy Bodies

Dementia with Lewy bodies (DLBs) is a neurodegenerative synucleinopathy characterized by fluctuating cognition, attentional and visuospatial deficits, recurrent complex visual hallucinations, and parkinsonian features typically occurring in the absence of resting tremor [217]. In patients exhibiting parkinsonism, reduced regional cerebral blood flow has been observed in M1 and left SMA [218].

Despite the clinical relevance of DLB, few TMS studies have investigated patients in moderate to advanced disease stages. Single-pulse TMS has demonstrated that rMT in DLB patients is similar to that of healthy controls [219]. However, in line with neurochemical studies demonstrating significant cholinergic deficits [220], SAI is significantly reduced in these patients [219,221].

Recent investigations aimed to clarify the neurophysiological basis of some hallmark symptoms, such as complex visual hallucinations. Notably, reduced SAI has been shown to correlate with the presence of hallucinations [221], highlighting the role of impaired cholinergic function in their pathogenesis. In addition, enhanced visual cortical excitability, reflected by lower phosphene thresholds, has been associated with greater hallucination severity [222], suggesting that both cholinergic dysfunction and altered occipital excitability may contribute to the clinical phenotype of DLB. In the context of DLB, rTMS has been primarily investigated for its effects on cognitive and neuropsychiatric symptoms. While some preliminary studies suggest potential benefits, the evidence remains limited and larger, controlled trials are needed.

Overall, while initial studies indicate that rTMS may offer therapeutic benefits for certain symptoms associated with atypical parkinsonian disorders, the current evidence base is limited by small sample sizes and methodological heterogeneity. Future research should focus on large-scale, randomized controlled trials to determine the efficacy, safety, and optimal protocols for rTMS in these complex and diverse conditions [223].

## 9. TMS in Secondary Parkinsonisms

While TMS and rTMS have been extensively studied for idiopathic PD, their application in secondary parkinsonism is significantly less documented and, therefore, direct evidence remains scarce. Secondary parkinsonisms, which can arise from different causes, such as vascular injury (vascular parkinsonism), drug exposure (drug-induced parkinsonism), head trauma (post-traumatic parkinsonism), infections, inflammation, toxins, or other underlying neurological disorders, often present different pathophysiological mechanisms and clinical features compared to idiopathic PD and atypical parkinsonisms [224]. In some cases, these forms may recover, at least partially, as soon as the underlying cause is removed or alleviated. This distinction is crucial, as the therapeutic effects of rTMS observed in idiopathic PD, often targeting motor cortex excitability and related neural circuits, may not be directly translatable in these cases.

### 9.1. Vascular Parkinsonism

Vascular parkinsonism (VP) is characterized by parkinsonian motor features resulting primarily from cerebrovascular lesions rather than neurodegenerative processes. VP often presents with lower body predominance, gait impairment, cognitive dysfunction, and poor response to dopaminergic therapy [225,226]. TMS has been employed to investigate cortical excitability and motor system integrity in VP, aiming to differentiate it from idiopathic PD and to understand its neurophysiology.

Several TMS studies have examined cortical excitability parameters in VP patients. Variable results in rMT in VP reflected a variable integrity of corticospinal pathways depending on the extent and location of vascular lesions [227,228]. Notably, cSP duration, an index of GABA-B mediated intracortical inhibition, has been found to be shortened in some VP cohorts, suggesting reduced intracortical inhibitory control possibly due to subcortical ischemic damage affecting motor networks [227].

Studies in VP and subcortical ischemic vascular dementia generally report that SICI remains unchanged or not significantly modified [225], suggesting that the functional integrity of these inhibitory connections is relatively spared compared to neurodegenerative parkinsonisms. However, in a study evaluating spinal ihibition, a reduction in SICI was observed [228]. Consequently, the differential pattern of SICI, markedly reduced in PD but preserved in VP, can serve as a neurophysiological marker to support the differential diagnosis between these clinical entities. Conversely, LICI appeared to be enhanced in a patient with VP [229], whereas ICF findings were similarly inconsistent, with reports ranging from normal to mildly altered facilitation profiles [228].

Furthermore, studies have also suggested altered sensorimotor integration in VP patients, as assessed by SAI. Reduced SAI in VP compared to controls indicates cholinergic dysfunction or impaired thalamocortical connectivity, which might contribute to gait and postural abnormalities characteristic of this condition [230].

Overall, TMS findings in VP support the notion of a disrupted balance between excitatory and inhibitory cortical circuits, secondary to subcortical ischemic injury. This neurophysiological profile contrasts with the more prominent cortical disinhibition seen in idiopathic PD, potentially offering a useful biomarker for differential diagnosis.

Therapeutically, VP is often refractory to standard dopaminergic therapy, prompting exploration of alternative treatments including neuromodulation techniques such as rTMS. Emerging evidence suggests that rTMS may improve motor symptoms and gait disturbances in VP patients by modulating cortical excitability and enhancing motor network function [231]. A recent systematic review assessed various interventions for VP, including rTMS. While levodopa response was generally modest in VP compared to idiopathic Parkinson’s disease, the review highlighted that rTMS and other novel therapies like vitamin D supplementation and photobiomodulation showed encouraging preliminary results [232]. However, these findings are limited by small sample sizes and risk of bias, underscoring the need for more rigorous trials to confirm efficacy and establish standardized treatment protocols.

In conclusion, although data remain limited, rTMS emerges as a promising non-invasive approach to modulate motor circuits in VP. However, future well-designed, large-scale studies are required to clarify its role and optimize stimulation parameters.

### 9.2. Other Secondary Parkinsonisms

While some research has explored rTMS for levodopa-induced dyskinesia in PD, which is a drug-induced movement disorder, this is distinct from drug-induced parkinsonism itself, and specific studies on rTMS for the latter are not prominent in the current literature. The duration of the post-excitatory inhibition after TMS was investigated in 16 patients with drug-induced parkinsonism and in 20 healthy control individuals. Notably, group comparison revealed a significant shorter post-excitatory inhibition in patients than in control individuals and regression analyses showed a negative correlation between the severity of the drug-induced parkinsonism and the duration of the post-excitatory inhibition [233]. Also, cSP was found to be abnormal both in patients with PD and in those with drug-induced parkinsonism. Namely, dopaminergic drugs modulated the cSP duration in patients and in normal subjects, through mechanisms acting mainly at basal ganglia and possibly also directly at cortical level [71].

Similarly, while TMS has been investigated in the context of traumatic brain injury (TBI), its specific efficacy in mitigating post-traumatic parkinsonian symptoms is not well-established. For other forms of secondary parkinsonism, such as those induced by toxins, infections, or as a manifestation of other neurological conditions (excluding vascular causes), there is a general lack of dedicated studies assessing the diagnostic and therapeutic potential of TMS. Therefore, although the principles of neuromodulation via TMS/rTMS offer a theoretical basis for early diagnosis and potential intervention, the current body of research does not provide robust evidence or specific guidelines for its application across the diverse spectrum of non-vascular secondary parkinsonisms. Clinical trials specifically targeting these other forms of parkinsonism are needed to determine the efficacy, optimal parameters, and specific indications for TMS and rTMS in these patient populations.

### 9.3. Functional Movement Disorders

TMS has been investigated also in functional movement disorders (FMDs), primarily as a tool to explore cortical excitability and understand pathophysiology, as well as a potential therapeutic modality. Studies have used TMS to assess motor cortical physiology in FMD, often revealing abnormalities in cortical inhibition and plasticity, though findings can be variable and sometimes differ from those in organic movement disorders [234]. For instance, some research suggests altered intracortical inhibition and facilitatory processes in patients with FMD, which might contribute to their symptoms. Repetitive TMS has also been explored, with some studies reporting symptomatic improvement in FMD, although the mechanisms remain under discussion, including the potential for placebo or non-specific effects alongside a “true” neuromodulation [235,236,237]. Overall, diagnostic and therapeutic utility of TMS in FMD is less established, though it can help in differentiating FMD from other movement disorders by demonstrating inconsistencies or normal physiological parameters where abnormalities would be expected in organic conditions.

## 10. From Neurophysiology to Precision Medicine: TMS as a Clinical Biomarker

To summarize, PD affects a complex multitransmitter system and network [238,239] characterized by a progressive nigrostriatal dopaminergic denervation that causes a dysfunction in the cortex-basal ganglia sensori-motor loops. A recent article summarized the anatomy and circuit models of the basal ganglia [239]: while neurochemical models define the basal ganglia pathology, the depletion of dopamine leading to excessive inhibitory output from the GPi/SNr to the thalamus, TMS could assesse the downstream consequences of this dysfunction on motor cortex excitability. The M1 indeed is the fundamental target from both the basal ganglia and cerebellum; the ventrolateral nucleus of the thalamus give inputs to M1 but also to SMA and premotor cortex. M1 projects back to the dorsolateral putamen and from M1 originate corticospinal pathways similar to that from the SMA and premotor cortex [240].

The loss of dopamine induces pathological beta-band oscillations (13–30 Hz) within the basal ganglia, particularly the STN, keeps the motor system in a state of tonic inhibition, effectively functional “locking” the circuit [241] and leading to circuit-level changes underlying the motor symptoms of PD. Moreover, several imaging studies suggest that, as neural loss progress in the basal ganglia, there is a loss of monoaminergic innervation in the cortex of PD [242,243,244] and a decreased activy of M1 [245]. Nevertheless, other studies showed an increased activation in M1 [246] in relation of particular phenotipes such as tremor, rigidity, or dyskinesia, as well as during the progression of the disease [247]. Accordingly, most single and paired-pulse TMS studies seem to be in agreement with the finding of motor cortex hyperexcitability [57,58,59]. Such finding might be the result of motor cortex reorganization to counteract the effect of low dopamine levels in the brain or reflects specific changes in cortical inhibitory and facilitatory systems.

Another possible explanation is that M1 iperactivity directly correlates with clinical variables, such as the severity of contralateral bradykinesia [57] or definite phenotypes as FoG [57,63]. In this regard, it is interesting to note that anomalous activity in the sense of hypoactivation of M1 has been found in patients with RBD before conversion to clinical PD [248]. At the same time, the GABAergic disinhibition obeserved is hypothesized to be an early, compensatory event initiated by the cortex to counteract the overall reduced drive, received from the hypokinetic basal ganglia loop [80,249]. TMS data indicate also an increase in excitability within a short-latency intracortical facilitatory circuit, which is regulated by glutamatergic neurotransmission. This specific hyperactivity is noted to be more pronounced in patients exhibiting LID and correlates with the severity of those involuntary movements [86].

However, this excitability appears “phenotype-dependent”: tremor-dominant patients consistently exhibit lower thresholds compared to the akinetic-rigid subtype [58], whereas patients experiencing FoG show a paradoxical increase in rMT during or just before freezing episodes [64]. Such discrepancies highlight a major methodological limitation: the lack of standardization regarding medication status (ON vs. OFF) and disease subtype, which complicates the use of MT as a standalone biomarker.

Basal ganglia are relevant to the sensori-motor integration during motor control [250,251,252]. In healthy individuals, the motor cortex “gates” sensory information, attenuating inhibitory effects during movement. In PD, this gating mechanism is distorted or lost, leading to significant impairments in movement accuracy and in the so-called “sequence effect” (i.e., the progressive decline in movement amplitude), characteristic of bradykinesia. The failure of these integration circuits is most effectively probed via SAI, the TMS paradigm that provides a direct neurophysiological measure of fast intracortical sensorimotor processing [28] and mainly mediated by colinergic pathways.

Furthermore, the multisystemic nature of this deficit involves the recruitment of secondary compensatory networks, such as the cerebellum. Early structural evidence from magnetic resonance imaging (MRI) supports this multisystem view: the volume of motor-dedicated anterior cerebellar regions initially increases in early PD before posterior, whereas non-motor regions may atrophy later in the disease. Another relevant point is the role exerted by the cerebellum in tremor pathophysiology, which is mediated by the cerebello–thalamo–cortical circuit. TMS integrates with this model by showing that changes in M1 excitability precede tremor onset and that the motor cortex is an active component of the tremor network [253].

The progressive degeneration of nigrostriatal dopaminergic neurons in PD leads to a substantial reorganization of the basal ganglia–thalamocortical circuitry, basically altering motor cortical plasticity [254]. In line with this level of dysfunction, TMS researche confirms this deficit and stratifies it as a stage-dependent phenomenon: while early-stage, drug-naïve patients may show exaggerated plasticity in the less affected hemisphere as a compensatory mechanism, advanced stages are characterized by a bilateral loss of the ability to modulate synaptic strength.

Finally, a critical insight involves the pathophysiology of LID, where TMS reveals a significant loss of depotentiation. In dyskinetic patients, cortical circuits fail to “erase” redundant motor information through homeostatic mechanisms, leading to the storage of aberrant motor patterns [255]. Furthermore, recent innovations in pairing STN-DBS with motor cortical TMS have demonstrated that associative plasticity can be artificially induced at specific latencies (approximately 3 ms and 23 ms), suggesting that neuromodulation can actively reshape dysfunctional neural networks and restore deficient plasticity [256].

Lastly, the consistent alterations in SICI and SAI across studies suggest that these metrics might serve as biomarkers for cortical excitability and GABAergic and cholinergic dysfunction. The translational potential of these TMS findings in PD lies in their ability to bridge the gap between structural imaging and clinical symptoms, although it still remains a challenge. While biomarkers from imaging or cerebrospinal fluid are essential for diagnosis, they are often poor indicators of day-to-day motor fluctuations or specific non-motor symptoms, like depression or cognitive decline. Conversely, some TMS measures, such as SICI and SAI, may provide in vivo and “real-time” functional readouts of GABAergic and cholinergic integrity, respectively. Future research should focus on “patient stratification”: for instance, using SAI to identify patients at higher risk of cognitive decline or gait disturbances, allowing for earlier and more targeted interventions.

On a therapeutic perspective, currently, HF-rTMS over M1 or DLPFC are the protocols closest to routine clinical translation, supported by large-scale meta-analyses and clear therapeutic effect sizes. Furthermore, TMS-derived measures of plasticity (e.g., PAS or iTBS) appear to be the most promising tools; e.g., identifying non-responders via a baseline TMS plasticity assessment would help clinicians and avoid ineffective treatments, thereby optimizing resource allocation in a stratified-medicine framework.

In this context, the integration of TMS with other modalities, such as high-resolution MRI and high-density EEG, will be crucial to bridge the gap between descriptive neurophysiology and a deeper understanding of the systems-level mechanisms underlying parkinsonian symptoms. Among them, the integration with resting-state functional MRI or wearable sensors for gait analysis will validate TMS markers as surrogate endpoints in neuroprotective trials, such as the ongoing NET-PD study [150].

**Figure 1:**
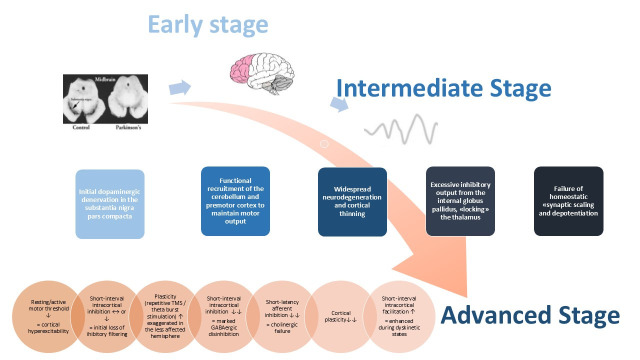
TMS markers across Parkinson’s disease progression: from early compensation to network breakdown and complications. ↓ = reduced/decreased; ↓↓ = markedly reduced/decreased; ↑ = enhanced/increased.

Figure 1 provides a schematic view of the PD from a TMS perspective.

## 11. Conclusive Remarks and Future Outlooks

Based on this narrative expert review, TMS has emerged as a promising non-invasive neuromodulatory technique in the context of PD, with growing evidence supporting its potential to alleviate both motor and non-motor symptoms. rTMS, particularly when applied to motor and prefrontal cortices, has demonstrated modest yet significant improvements in symptoms such as bradykinesia, gait disturbances, depression, and cognitive dysfunction. However, heterogeneity in stimulation protocols regarding frequency, intensity, target area, and number of sessions continues to limit comparability across studies and hinders the establishment of standardized therapeutic guidelines.

To fully capture the complexity of PD pathophysiology, TMS findings must be integrated into a broader network physiology framework. Recent advances in multimodal approaches, such as combined TMS-high-density EEG and TMS-functional MRI, have begun to elucidate how local cortical abnormalities resonate across distant neural nodes. For instance, while paired-pulse TMS identifies local GABAergic deficits, TMS-EEG studies reveal that these are often associated with pathological beta-band (13–30 Hz) hypersynchrony and altered long-range connectivity within the BGTC loops.

Integrating TMS with resting-state functional MRI has shown that impaired cortical plasticity (measured via PAS or TBS) correlates with reduced functional connectivity between the M1 and the basal ganglia, as well as with compensatory recruitment of the cerebello-cortical networks. These network-level disturbances suggest that the clinical manifestations of PD are not the result of focal lesions but rather the effect of a systemic failure in neural communication. Adopting such a multimodal perspective is essential for developing “network-based” neuromodulation strategies that target the distributed circuits underlying complex symptoms, such as the FoG or cognitive decline.

The clinical translation of TMS in parkinsonian disorders faces significant methodological hurdles. A major limitation is the lack of standardization in coil positioning, where manual placement often leads to higher variability compared to MRI-guided neuronavigation. Furthermore, stimulus intensity protocols vary significantly between studies, complicating direct comparisons. Another critical factor limiting the clinical impact of rTMS is the “one-size-fits-all” approach. Emerging evidence suggests that tailoring stimulation parameters (e.g., frequency, burst patterns, and anatomical targeting based on individual structural MRI) is essential to overcome the high inter-individual variability observed in PD patients. This is particularly evident in atypical and secondary parkinsonisms, wehere a “TMS-bases” evidence is still preliminary and, at this stage, does not support a standardized clinical application yet.

From a therapeutic perspective, although statistically significant improvements in UPDRS-III scores are frequently reported, the clinical meaningfulness (effect size) remains modest in many trials. The presence of null findings or results non-superior to sham stimulation, particularly in complex symptoms like gait, must be acknowledged. Future research should prioritize large-scale, multicenter trials with standardized dosing to move beyond pilot-level evidence.

Beyond standard protocols, a significant shift in recent years involves the move toward individualized stimulation parameters. Fixed-target approaches often overlook the high inter-individual variability in cortical excitability and anatomical atrophy present in PD. Tailoring stimulation based on individual motor thresholds or MRI-guided neuronavigation is essential to optimize clinical outcomes. Furthermore, the cerebellum has emerged as a crucial node within the complex parkinsonian network. Recent evidence suggests that cerebellar rTMS can effectively modulate the cerebello–thalamo–cortical pathway, offering a novel therapeutic window for managing LID and postural instability, which are often refractory to conventional drugs and cortical stimulation protocols.

To conclude, despite its growing value as a tool linking network pathophysiology to targeted neuromodulation, the clinical impact of TMS in PD remains constrained by methodological heterogeneity and the need for standardized, phenotype-driven and multimodal approaches. Once optimized, however, TMS will represent a valuable translational bridge between network-level pathophysiology and precision neuromodulation in PD and other movement disorders.

## Figures and Tables

**Table 1:** Summary of transcranial magnetic stimulation (TMS) parameters, neurobiological mechanisms, and clinical evidence in Parkinson’s disease (PD).

TMS Measure	Neurobiological Mechanism	Key Findings in Parkinson’s Disease	Clinical/Translational Utility	Level of Evidence
**SAI** (short-latency afferent inhibition)	Cholinergic pathways and sensorimotor integration	Significantly reduced in patients with cognitive impairment	**Prognostic biomarker:** high sensitivity for predicting cognitive decline and dementia risk	**High** (clinically relevant)
**CSP** (cortical silent period)	GABA receptor-mediated inhibition (cortical component)	Typically shortened in “OFF” state; often normalized by dopaminergic medication.	**Monitoring tool:** reliable index of motor cortical inhibition and response to therapy	**High** (clinically relevant)
**SICI** (short-interval intracortical inhibition)	GABA receptor-mediated inhibition	Consistently reduced in early patients; helps distinguish Parkinson’s disease from some secondary parkinsonisms	**Research/diagnostic biomarker:** reflects cortical disinhibition; potential for patient stratification	**High** (consistent across studies)
**LICI** (long-interval intracortical inhibition)	GABA receptor-mediated inhibition	Generally reduced, though results are more heterogeneous than SICI	**Research tool:** investigates long-latency inhibitory circuits; reflects disease progression	**Moderate** (contradictory findings reported)
**rMT** (resting motor threshold) **MEP** (motor-evoked potential) **amplitude**	Cortico-spinal excitability	Often normal or slightly increased (rMT reduced); highly dependent on medication (ON/OFF)	**Monitoring tool:** assesses global motor cortex excitability; useful in longitudinal studies	**Moderate** (high inter-individual variability)
**PAS** (paired associative stimulation)	NMDA-dependent activity. Long-term potentiation (LTP)-/long-term depression (LTD)-like plasticity	Reduced plasticity in Parkinson’s disease; restoration of PAS response correlates with clinical benefit	**Mechanistic biomarker:** assesses synaptic plasticity; useful for evaluating disease progression	**Moderate/high** (clinically relevant)
**rTMS/TBS** (repetitive TMS/Theta burst stimulation)	Long-term potentiation (LTP)-/depression (LTD)-like plasticity	Variable results; therapeutic potential for motor symptoms and levodopa-induced dyskinesia	**Therapeutic intervention:** high potential but requires standardization of protocols (coil/intensity)	**Variable** (Level A for motor symptoms in some guidelines)

**Table 2:** Comparative neurophysiological signatures and diagnostic utility of TMS across parkinsonian syndromes.

Disease	TMS Changes	Mechanistic Interpretation	Clinical/Diagnostic Value
**Parkinson’s disease (PD)**	↓ SICI, ↓ SAI (late), ↓ PAS/TBS response	**Basal ganglia–thalamo–cortical loop disruption:** cortical disinhibition due to BG-thalamo–cortical imbalance; impaired synaptic plasticity.	Marker of disease progression and dopaminergic responsiveness.
**Progressive supranuclear palsy (PSP)**	↓↓ SICI, ↓ CBI, paradoxical response to continuous TBS	**Frontal-cerebellar breakdown:** direct degeneration of premotor areas and dentato–thalamo–cortical pathways; loss of homeostatic plasticity.	Differential diagnosis from PD; reflects high fall risk and axial rigidity.
**Multiple system atrophy (MSA)**	↓ SICI (MSA-P), ↓ SAI, absent TBS plasticity	**Widespread connectivity failure:** disruption of both extrapyramidal and cholinergic systems; early “freezing” of synaptic plasticity.	Distinguishes MSA-P from MSA-C; identifies severe autonomic/cognitive phenotypes.
**Corticobasal degeneration (CBD)**	↑ rMT (asymmetric), ↓ iSP, expanded motor maps	**Focal cortical hypoexcitability:** severe focal atrophy of the primary motor cortex/premotor areas; breakdown of transcallosal coordination.	Signature of focal cortical degeneration; neurophysiological marker for “alien limb” and apraxia.
**Dementia with Lewy bodies (DLB)**	↓↓ SAI, ↓ phosphene threshold	**Cholinergic failure:** massive loss of top-down cholinergic modulation (nucleus basalis of Meynert); occipital hyper-excitability.	Biomarker for cognitive fluctuations and risk of visual hallucinations.

*Legend:* CBI, cerebellar brain inhibition; iSP, ipsilateral silent period; MSA-C, multiple system atrophy with cerebellar features; MSA-P, multiple system atrophy with predominant parkinsonism; PAS, paired associative stimulation; rMT, resting motor threshold; SAI, short-latency afferent inhibition; SICI, short-interval intracortical inhibition; TBS, theta burst stimulation. ↓ = reduced/decreased; ↓↓ = markedly reduced/decreased; ↑ = enhanced/increased.

## Data Availability

No new data were created or analyzed in this study. Data sharing is not applicable to this article.
